# Testing an implementation strategy bundle on adoption and sustainability of evidence to optimize physical function in community-dwelling disabled and older adults in a Medicaid waiver: a multi-site pragmatic hybrid type III protocol

**DOI:** 10.1186/s13012-019-0907-1

**Published:** 2019-06-13

**Authors:** Sandra L. Spoelstra, Monica Schueller, Alla Sikorskii

**Affiliations:** 1 Kirkhof College of Nursing, Grand Valley State University, 301 Michigan St, Room C352, Grand Rapids, MI 49504 USA; 2 Departments of Psychiatry and Statistics and Probability, Michigan State University, East Lansing, USA

**Keywords:** Adoption, Sustainability, Implementation, Implementation strategies, Physical function, Community-dwelling, Older adults, Medicaid waiver, Study protocol, Randomized controlled trial

## Abstract

**Background:**

In partnership with a state Medicaid home and community-based waiver program, this study tests implementation strategies for adoption and sustainability of an evidence-based intervention to support disabled and older adults who have difficulty with physical function and daily living tasks. A multi-level implementation strategy bundle will be directed at relationship, coalition, and team building; readiness to implement, leadership, and clinician attitude toward evidence assessments; intervention and facilitation training; interdisciplinary coordination; facilitation; and audit and feedback to support practice change.

**Methods:**

Knowledge-to-Action model underpins this 2-arm, 3-year pragmatic mixed method randomized hybrid type III trial in 18 waiver program sites in Michigan. Data will be collected on sites, 775 clinicians (registered nurses, occupational therapists, social workers), and 15,000 disabled and older adults. Consolidated Framework for Implementation Research guides examination of site, clinician, and beneficiary characteristics; clinician attitude and self-efficacy; leadership and readiness to implement; and intervention impact on beneficiary outcomes. Sites will be randomized to either usual waiver care with internal facilitation of the bundle of implementation strategies or usual waiver care with both internal and external facilitation of the bundle. Primary outcomes are site-level adoption and sustainability over 12 months, and intervention effects on these outcomes are hypothesized to be mediated by clinicians’ attitude and self-efficacy. At the beneficiary level, by addressing the individual’s capabilities and home environment, the intervention is hypothesized to improve secondary outcomes of activities of daily living, pain, depression, falls, emergency department visits, and hospitalizations. Baseline site readiness and leadership and stages of implementation at 6 months will be explored as potential moderators. Linear mixed effects models will be used to test intervention effects on primary outcomes, with bias-correcting analytic strategy in mediation analyses. Generalized linear mixed effects modeling will be employed for the analysis of intervention effects on secondary outcomes.

**Discussion:**

Synthesizing findings within and across the sites, we will specify how leadership, readiness for change, and level of facilitation enhance capacity for adoption and sustainability of an evidence-based intervention in an under-resourced Medicaid setting that cares for disabled and older adults.

**Trial registration:**

ClinitalTrials.gov, NCT03634033. Registered 16 August 2018.

**Electronic supplementary material:**

The online version of this article (10.1186/s13012-019-0907-1) contains supplementary material, which is available to authorized users.

Contributions to the literature

- This trial will provide immediate impact on the waiver by providing access to an evidence-based intervention that improves physical function and the ability to perform daily tasks, and assists disabled and older adults to remain living in the community.
- A long-term impact will be to enhance the waiver’s capacity for practice innovations through development and testing of a bundle of implementation strategies to enhance adoption and sustainability of evidence-based interventions.
- The trial will specify stages of implementation completion and how leadership, readiness for change, clinician attitude, and self-efficacy enhance capacity for practice improvement and adoption of evidence-based programs.

## Background

There are 962 million people aged 60 and older worldwide [1], and 42% in the USA report problems with physical function, which can lead to difficulty with daily tasks and nursing home placement [2–5]. Interventions that optimize physical function to support aging-in-place are needed, particularly in settings that care for disabled and older adults. We propose to address this urgent need through the testing of an implementation strategy bundle on adoption and sustainability of an adapted intervention [6] that improves adults’ ability to conduct daily tasks [7–10]. The implementation strategy bundle includes relationship, coalition, and team building; readiness to implement, leadership, and clinician attitude toward evidence assessments; intervention and facilitation training; interdisciplinary coordination; internal and external facilitation (IF, EF); and audit and feedback. Multi-level approaches are known to support incremental single strategy effects, as well as a synergistic effect from all the strategies [11]. Thus, we test two topics of import to the field of implementation science - adoption and sustainability [12–16] - and explore mechanisms of action [17] to support uptake, while implementing a complex multi-component intervention in a disabled aging population. Real practice change and improved outcomes will not occur without adoption and sustainability [13].

### Model guiding implementation

**Fig. 1:**
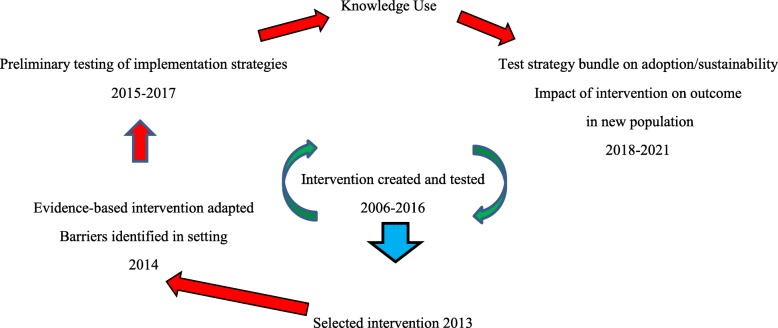
Applying the Knowledge-to-Action model to the intervention creation, testing, setting/population, adaptation, barriers, and knowledge use

The Knowledge-to-Action [18, 19] model underpins our approach (see Fig. 1) to implementation. Evidence was created and efficacy established by others [7–10]. In prior work, the evidence-based intervention was selected, barriers to implementation were identified, and the intervention was adapted to the setting and population [6]. This study will evaluate adoption and sustainability of the intervention and beneficiary outcomes.

## Methods

The overall goal of the study is to implement evidence in a waiver program to improve the ability of disabled or older adults to perform daily tasks. Objectives for this study are to test the deployment of an implementation strategy bundle on adoption and sustainability of the intervention and to examine the effect of the intervention on beneficiary outcomes.

### Aims

*Aim 1:* To test the effects of an implementation strategy bundle with IF alone versus an implementation strategy bundle with IF + EF with respect to the site-level outcomes of adoption and sustainability (primary) and beneficiary-level outcomes of activities of daily living/instrumental activities of daily living (ADL/IADLs), pain, depression, falls, emergency department (ED) visits, and hospitalizations (secondary) over the next 12 months.

*Aim 2 mechanism-of-action*: To determine whether the effects of IF + EF versus IF on primary outcomes are mediated by clinician attitude or self-efficacy at 9 months.

*Aim 3:* To benchmark the effects of IF and IF + EF on beneficiary outcomes following implementation as compared to pre-intervention.

*Exploratory aim 4*: To compare the primary and secondary outcomes within 12 months, potential mediators at 9 months, and baseline leadership and readiness for sites with SIC of > 50% versus sites with SIC of < 50% at 6 months in each arm.

*Exploratory aim 5*: To explore whether baseline site leadership and readiness moderate the impact of IF + EF compared to IF on primary and secondary outcomes within 12 months in order to determine which sites may require facilitation that is more intensive.

*Exploratory aim 6*: To evaluate clinician satisfaction at 1 month and the cost of implementation and policy impact for IF and IF + EF at 12 months.

### Study design

Testing in this study will occur in a 2-arm, 3-year pragmatic hybrid type III [20, 21] mixed method randomized trial design [22–24]. Hybrid III trials examine implementation strategy effect (primary) while gathering data on the intervention outcomes (secondary) [21].

*Arm 1* includes usual waiver care and an implementation strategy bundle with IF.

*Arm 2* includes all components of Arm 1 plus additive EF.

#### Setting

The setting is 18 Medicaid home and community-based (MI Choice) waiver sites in Michigan. The waiver supports low-income, nursing home eligible, disabled, and older adults in the community, providing 19 services (e.g., personal care and meals) through registered nurse (RN) and social work (SW) case management.

#### Participants, recruitment, contracts, and consents

**Fig. 2:**
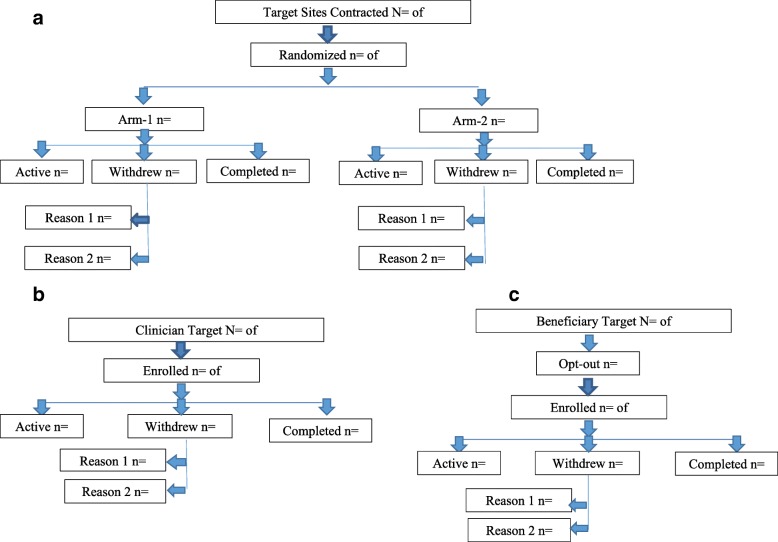
CONSORT-like figure of recruitment and participant status to be tracked. **a** Site CONSORT-like figure. **b** Clinician study status CONSORT-like figure. **c** Beneficiaries study status CONSORT-like figure

We have three levels of participation: the site, the clinician, and Medicaid beneficiary, with individualized recruitment plans for each. Figure 2 shows a CONSORT-like flow chart of recruitment by site, clinician, and beneficiary.

*Sites*. We recruited 18 waiver sites in Michigan, have letters of support, and anticipate all will be contracted.

*Clinicians*. We will recruit clinicians via a flyer sent by email. In addition, the IF at each site will encourage clinicians to complete the training and use the intervention by providing verbal and written prompts during routine communications (email or face-to-face) and at staff meetings. We expect 80% (620 of 775) of clinicians employed at the sites to consent electronically prior to online training, complete the training, and use the intervention with beneficiaries.

*Beneficiaries*. The trained clinicians will recruit beneficiaries during a home visit using an algorithm that was designed during prior work and a cognitive aid on a pocket card that will be provided during training. The algorithm is a set of rules the clinician follows to use critical thinking when examining the beneficiary’s need or potential benefit from the intervention. We expect 60% (9,000 of 15,000) of beneficiaries to not opt-out and receive the intervention.

#### Inclusion criteria

*Sites*. Eighteen sites under contract by the Michigan Medicaid program which provide waiver care and utilize an electronic health record (EHR) from EHR company are included. Excluded are 2 sites which do not use the EHR.

*Clinicians*. Clinicians who are employed by the sites who consent and are trained in use of the intervention are included. Excluded are clinicians who do not consent and/or do not complete the training.

*Beneficiaries*. Beneficiaries who do not opt-out and who may need and benefit from the care will receive the intervention. Excluded are beneficiaries who opt-out.

#### Evidence-based intervention

The evidence-based intervention reduces the effect of problems with physical function among low-income older adults living in the community by addressing an individual’s capabilities and the home environment [7–10]. The intervention is a 16-week structured program delivered by occupational therapists (OTs) who conduct 6 home visits and provide assistive devices, RNs who conduct 4 home visits, and a handyman who provides home alterations (e.g., installs devices, environmental modifications, and home repair). The team provides consultation with older adults to help the individuals identify daily activity goals (e.g., taking a shower and walking to the bathroom), evaluates barriers to achieving those goals, and attains outcomes collaboratively. OTs assist older adults to carry out ADLs, IADLs, and discretionary activities that are challenging at home such as functional mobility, meal preparation, bathing, and dressing. RNs target underlying issues that influence ADLs and IADLs, such as pain reduction, improvement in mood, fall prevention, incontinence management, and medication review and management with the help of a pharmacist. In prior work, the intervention was adapted [6] to fit the waiver setting and population: SWs were added to address social and emotional needs; the number, type, and timing of clinician visits were tailored to the beneficiary’s need; RNs were trained in medication management; and training and documentation modes were modified to fit the setting.

#### Guiding framework

**Fig. 3:**
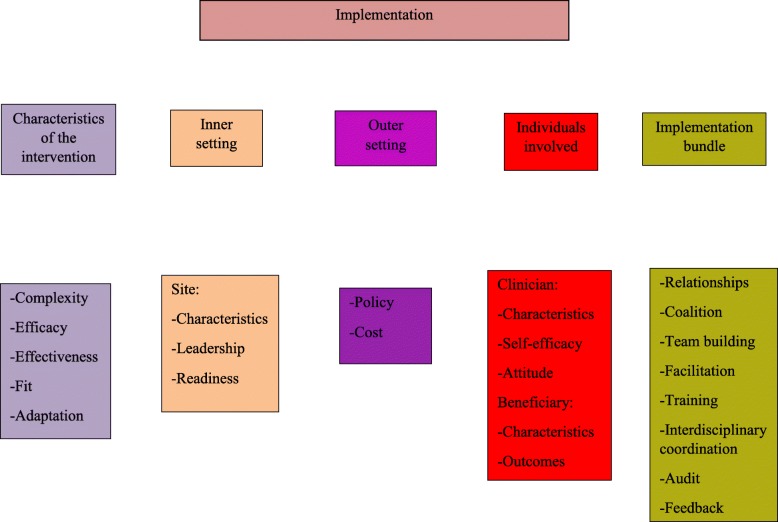
Consolidated Framework for Implementation Research (CFIR) factors influencing implementation strategy bundle and outcomes

Consolidated Framework for Implementation Research [25, 26] supports our approach to practice change (see Fig. 3). We will examine characteristics (site, clinician, and beneficiary), clinician attitude and self-efficacy, inner setting [27] leadership and readiness to implement, and outer setting [28] policy, as each may impact adoption and sustainability.

#### Implementation strategies

**Fig. 4:**
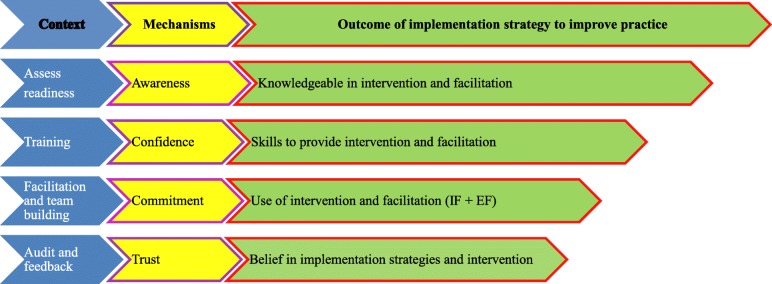
Relationships among mechanisms-of-action context, mechanism category, and expected outcome of implementation strategy

We will deploy a 9-component implementation strategy bundle (see Table 1). As few studies focus on sustainability of implementation efforts in knowledge translation during implementation, we examine the mechanisms of action of awareness, commitment, confidence, and trust [17] during implementation (see Fig. 4). Materials; procedures; providers; mode, where provided; duration; intensity and dose; and tailoring, if needed, for each strategy are presented. We also delineate which strategies we expect to trigger the mechanisms of action.

**Table 1:** Implementation strategies deployed, domains affected, definition, actors, actions/target of actions, temporality, dose, and outcome affected

Domain	Organization readiness	Build IF coalition	IF training	Clinician training	Clinical teams	EF centralize oversight	Audit and feedback implementation intervention fidelity
Definition	Aspects of an organization determine readiness to implement	IFs coalition to share implementation knowledge	Dynamic interactive training via varying learning methods and supervision focused on implementation	Training to conduct intervention	Develop/implement teams of clinicians who meet, reflect, and share learnings	Makes things easier for others: support to change attitudes, habits, skills, way of thinking and working	Collect and summarize clinical performance and monitor, evaluate, and modify clinician behavior
Actors	PI, PM, waiver clinician	IF, research team	Research team for IF	IF for clinicians	IF, clinicians	EF	PI, PM, IF, EF
Actions	Administer tools, analyze results	Online Bb forum to build capacity, share best implementation strategies	Identify and train train-the-trainers/IF	Train clinicians, post-test, remediate	IF leads interdisciplinary coordination, feedback on implementation/intervention	Assistance to IF (arm 2)	Monitor Bb and EHR, SIC (scorecard)
Target of action	Site	IFs	IFs	Clinicians	Clinicians	IF	Clinicians
Temporality	Before implementation		When starting implementation			1 month after implement	1 week after intervention and ongoing
Dose	Surveys completed baseline	1 h discussion monthly	1.5 h Bb online training; remediate prn	5.5 h online Bb training	Meet 15 min per month per beneficiary cared for	Weekly for 30 min to 1 h until issues resolved	Weekly results to scorecard in Bb and IF reviews with clinicians, EF reviews with IF
Outcomes affected	Acceptability, readiness	Acceptability	Adoption, sustainability			Adoption, sustainability	Adoption, sustainability, outcomes

##### Strategy 1

We will form both informal and formal relationships to conduct this study. We expect this strategy to prompt the mechanisms of action of trust (see Fig. 4).

*Informal relationships.* Informal relationships were built between the sites, the investigator, and the state policymakers during 20 years of prior work. During this study, the investigator will continue to interact with the policymakers on a monthly basis, updating on study progress via a brief email (< 120 words); with the sites on a quarterly basis, providing a 1-h continuing education unit (e.g., incontinence, pain management); and with both by attending the 1-h face-to-face meeting when requested.

*Formal relationships*. For this study, an agreement, memorandum of understanding, or contract (site and university, state and university, and EHR company and university) will be executed prior to data collection and last for the duration of the study. The roles and responsibilities of each partner (site, investigator, state, and EHR company), a data use agreement, and a timeline for the study will be delineated. A key component of the success of the study is the IF. Delineated in the contract is the selection of the IF by the investigator with guidance by the site managers. The IFs are supervisors/employees that work for the waiver site and will conduct facilitation at their site as “Champions.” In addition, we will utilize a waiver employee as the EF, who was an early adopter of the intervention in prior work, as a “Super-Champion” for arm 2.

##### Strategy 2

Data on readiness to implement, to prompt the mechanisms of action of awareness (see Fig. 4), and leadership, to examine the need to do intermittent booster training for facilitators, will be collected from clinicians in an online 10-min survey distributed prior to training. Within the readiness assessment, we focus on subscales of training, pressure for change, and attributes of growth, efficacy, and influence. Within the leadership assessment, we focus on subscales of proactive, knowledgeable, supportive and perseverant leadership.

##### Strategy 3

Data on clinician attitude and self-efficacy will be collected in an online 10-min survey distributed prior to training and at 9 months to examine changes over time. In the self-efficacy assessment, we examine if the training prompts improvement. In the attitude assessment, we focus on openness, appeals of the new intervention, willingness to using the intervention, and conflict between clinical experience and research results (divergence). We expect this strategy to prompt the mechanisms of action of confidence during implementation (see Fig. 4).

##### Strategy 4

Clinicians will be trained in the intervention, and supervisors (IFs and EF) will be trained in facilitation. We expect this strategy to prompt the mechanisms of action of confidence during implementation (see Fig. 4).

*Clinicians*. RNs, SWs, and OTs will complete a 5.5-h, 20-module, 6-article online training program prior to providing the intervention to beneficiaries. Clinicians are expected to complete the training in its entirety within a 30-day time period and will receive a continuing education certificate upon completion. Modules include training on person-environment fit, person-centered care, and building collaborative relationships and self-efficacy. In addition, other modules include readiness-to-change, motivational interviewing, cognitive behavioral therapy, problem solving, brainstorming, therapeutic communication, reflection, and interdisciplinary coordination. An additional file shows this in more detail (see Additional file 1). The role of each discipline and care to be provided are covered, with in-depth medication management instructions, a case study, and handouts to use during care. A pocket card cognitive aid (see Additional file 2) will be provided to assist clinicians in tailoring care to the beneficiary’s needs and desires. We may need to tailor and/or boost education to a specific site and/or group of clinicians on use of the intervention, with brief (20–30 min) online training modules, after we examine fidelity to the intervention monthly (months 2 to 12).

*Facilitators*. IFs and EF will complete a 1.5-h, 10-module online training program on facilitation. Modules for the IF include evidence supporting implementation strategies, the facilitator role, quality improvement, problem solving, how to provide feedback, use of reflection, counseling, motivational interviewing, and remediation. An additional file shows this in more detail (see Additional file 3). An implementation toolkit will be provided, and the plan will be reviewed. The module for the EF includes a train-the-trainer approach for facilitation of IFs. We may need to tailor and/or boost education with some or all of the IFs if data on implementation strategy fidelity show implementation is not occurring as expected.

##### Strategy 5

IFs will form a coalition to support implementation. Monthly for 1 h for 12 months, the IFs will have an online collaborative meeting hosted by the investigator. The purpose of the coalition is to build capacity among the IFs by sharing best practice use of the implementation strategies. The investigator will provide feedback on the number of clinicians trained and fidelity to implementation strategies and the intervention. Discussion will then occur among the group on use of implementation strategies to improve implementation. We expect this strategy to prompt the mechanisms of action of commitment during implementation (see Fig. 4).

##### Strategy 6

IFs will prompt and lead intermittent interdisciplinary coordination among the clinicians (RN, SW, and OT), as needed, to promote teamwork, brainstorming, and problem solving to support beneficiary goal attainment. We expect interdisciplinary coordination will occur via phone, email, and/or face-to-face for 15 min per month for each beneficiary cared for. However, this is dependent upon the complexity of the beneficiary’s care and is tailored in time and intensity based on the needs of the person. We expect this strategy to prompt the mechanisms of action of commitment during implementation (see Fig. 4).

##### Strategy 7

IFs will facilitate clinician implementation at each site in arm 1 and 2, and the EF will facilitate IFs in arm 2 tailored to a site’s needs. We expect that an IF will spend between 2 to 5 h a week per month (months 2 to 12) facilitating implementation via face-to-face, email, or phone. Also, we expect that an IF will spend 1 h a month for 12 months interacting with IFs from other sites to learn best practices for implementation. Once the clinicians are trained and using the intervention with the beneficiaries, the IFs will be absorbed within usual IF duties. IF actions include training, coaching, consultation, supervision, modeling, problem solving, and providing feedback, supporting, instructing, demonstrating, and assisting with evaluation. We will use an audit tool with these actions to examine facilitation monthly (months 2 to 12) (see Additional file 4). We expect this strategy to prompt the mechanisms of action of commitment during implementation (see Fig. 4).

##### Strategy 8

An audit of implementation strategy and intervention fidelity (see Additional file 5) with feedback will occur to assist IF’s work with clinicians in arms 1 and 2. The audit will be conducted by the study team, and feedback will be provided to the IFs weekly via email and reviewed monthly during the online coalition meeting for both arm 1 and 2. We expect this strategy to prompt the mechanisms of action of confidence, commitment, and trust during implementation (see Fig. 4).

##### Strategy 9

The EFs work with and support the IFs in arm 2. Feedback will be provided to the EF weekly via email by the study team, with a request to provide external facilitation as needed when implementation is not occurring as expected. Similar to IF actions, EF actions include training, coaching, consultation, supervision, modeling, problem solving, and providing feedback, supporting, instructing, demonstrating, and assisting with evaluation. We will use an audit tool with these actions to examine facilitation monthly (months 2 to 12) (see Additional file 4). We expect this strategy to prompt the mechanisms of action of commitment during implementation (see Fig. 4).

#### Measures

In this study, we will measure site, clinician, and beneficiary level data (see Table 2). Site measures include characteristics, leadership, readiness to implement, and implementation completion. Clinician measures include characteristics, attitude toward evidence-based care, self-efficacy, and training completion. Beneficiary measures include characteristics and outcomes. We also measure fidelity to implementation strategies and the intervention. Measures are shown in Table 2, and the data collection plan is shown in Table 3.

**Table 2:** Measures deployed in study by domain, construct, concept, aim, who/how measured, and who collects

Framework	Construct	Concept(s)	Aim(s)	Who measured	How measured	Collector
Inner setting	Readiness for Implementation	Leadership engagement	5	Sites	ILS	Project manager
		Readiness to implement			ORC	
		Size of site waiver agency			Administrative data 2018	–
		Quality scores			Quality data	
Individual:site and person	Demographics	Site: number of employees/beneficiaries, quality scores, cost	–	Sites	Administrative data 2018	
		Clinician: age, race, sex, discipline, degree, years of experience as clinician and in WA		Clinician	Survey	Project manager
		Beneficiary: age, sex, race		Beneficiary	MDS-HC	–
	Clinician: engagement	Attitude, self-efficacy	2	Clinician	1. EBPAS 2. GSE	Project manager
	Beneficiary outcomes	ADLs, IADLs falls, pain, depression/ED/hospital	3 and 4	Beneficiary	MDS-HC	–
Process	Training	Knowledge with intervention	2	Clinician	Survey	Project manager
		Knowledge with IF/EF		IF/EF		
	Team building	Interdisciplinary coordination		Beneficiary	EHR progress notes	–
	Coalition building	Occurrence/type		IF	Data tool	–
Inner setting	Adoption	Fidelity to training	1 and 2	Clinician	Type, date, number done	Project manager
		Fidelity to IF/EF		IF/EF	IF data tool	Research assistants
		Fidelity to intervention		Beneficiary/patient	EHR care plan	–
	Sustainability	Fidelity to change		Sites	SIC	Research assistants
	Acceptability	Satisfaction with training	6	Clinician IF EF	Survey	Project manager
	Cost	Dollars expended	6	Clinician IF EF beneficiary	Wages, benefits	Project manager
Outer setting	Policy	Payment for incentive	6	CMS contract	Contract 1 October 2022	Project manager

**Table 3:**
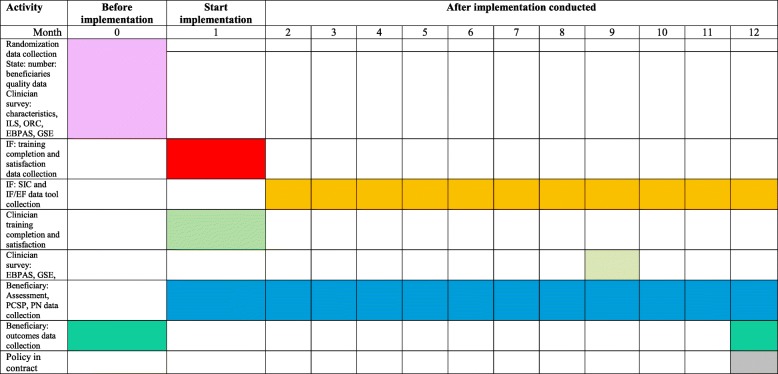
Data collection activity, month, where obtained from (state, clinicians, IF, EF, EHR), and what obtained

##### Characteristics

Characteristics will be collected at baseline. Site characteristics include size; quality scores; number of supervisors, clinicians, and beneficiaries; and years in the waiver. Clinician characteristics include age, race, gender, discipline, and years working at waiver program site. Beneficiary characteristics include age, gender, and race.

##### Tools

We use 6 tools in this trial. Implementation Leadership Scale (ILS) is a 12-item, 4-subscale (Cronbach .93–.97) tool that assesses leadership behaviors and actions and will be collected from clinicians at baseline [29, 30]. Organizational Readiness for Change (TCU-ORC) is a 124-item tool with subscales (needs of clinician, program, training, pressure for change, resources, and attributes) and 3-open ended questions and will be collected at baseline from clinicians (Cronbach .88) [31]. Evidence-Based Practice Attitude Scale (EBPAS) is a 50-item tool with 12 subscales with 4 constructs (openness, appeal, requirements, and divergence) and will be collected from clinicians at baseline and 9 months [32]. General Self-Efficacy (GSE) is a 10-item tool (Cronbach’s alpha .79–.90) and will be collected from clinicians at baseline and 9 months [33]. Minimum Data Set-Home Care (MDS-HC) is a self-reported, person-centered assessment for the collection of beneficiary minimum essential nursing data, with reliability and validity, and used in the waiver since 1993 and will be collected in the EHR as part of usual care. We obtain variables for our study from the MDS-HC [34]. Stages of Implementation (SIC) is an 8-stage tool that examines completion of activities during implementation, adoption, and sustainability and will be collected from the IFs monthly (months 2 through 12) [35, 36].

##### Fidelity

We will examine fidelity to the implementation strategies and the intervention.

*Fidelity to the implementation strategies*. An implementation strategy fidelity checklist measures number of consented clinicians, number of completed readiness and leadership assessments, number of clinicians trained, number of IF actions taken, and number of interdisciplinary coordination meetings and will be collected monthly (months 2 through 12). Based on a review of the literature, IF actions will be coded as training, coaching, consultation, supervision, modeling, problem solving, providing feedback, supporting, instructing, demonstrating, and assisting with evaluation. Clinician and facilitator training acceptability will be evaluated by number of clinicians and facilitators who completed training. Costs of implementation will be estimated based on the time spent training and increased number of home visits. Policy impact will be examined in the waiver contract.

*Fidelity to the intervention*. An intervention fidelity checklist will be used to examine the EHR for those beneficiaries who consent and receive care from trained clinicians. This will include OT assessments at baseline; SW mood assessment, as needed; and RN medication reviews and will be collected monthly (months 2 through 12). In addition, within the person-centered service plan, we will examine the presence of the beneficiary’s desires and needs being documented. Within progress notes documentation, we will examine use of brainstorming, problem solving, and role modeling.

##### Outcomes

Primary outcomes are implementation strategy impact on adoption and sustainability of the intervention. Stages of implementation completion will be derived via 3 SIC scores. First, the number of stages completed is a simple count of progression through stages; the score is the last stage in which at least one activity was performed. Second, time spent in each stage is calculated by taking the difference between the date of completion of the first activity in the stage and the date of completion of the last activity in the same stage. Skipped activities are not included. If a site skips the last activity in a stage and completes an activity in a subsequent stage, they automatically move to the subsequent stage. However, if they later complete the skipped activity, the duration score is adjusted for the original (earlier) stage to include the activity. Third, the date of completion of all stages is logged within stage 8. For sites that choose to discontinue implementation at any point in the process, the date is logged in the furthest stage that the site enters. In the case where data are summarized before the stage is complete but a site has not discontinued implementation, the site data are treated as being censored, just as it would in a standard time-to-event or survival analysis [37]. Proportion of activities completed is calculated as the number of activities completed divided by the number of possible activities in each stage. Activities in each stage are ordered based on their logical progression up to the last activity the site completes in the stage or completion of the final activity in the stage. Achievement of either activity indicates completion of that stage. Secondary outcomes are intervention effects on beneficiary ADLs, IADLs, pain, depression, falls, ED visits, and hospitalizations.

#### Sample size and power calculation

Site sample size was based on the number of available sites, with 9 randomized to each arm. Assuming correlation of 0.7 between pairs of 11 repeated measures, the adjusted effect size of 1.2 was detectable with power of 0.80 or greater in two-sided tests at 0.05 level of significance. The effect size is expressed as Cohen's d, difference between trail arm means in standard deviation units. If observed differences are smaller than 1.2 of the standard deviation, statistical significance will not be reached, then the estimate of the effect size will be obtained. For beneficiary outcomes, we assumed 750 beneficiaries per site and conservative intraclass correlation coefficient of 0.01 to obtain the design effect factor of 8.49. In the comparison of the trial arms, the adjusted sample size is 795 per trial arm, which allows to detect effect size as small as 0.14. As larger effects were seen in the prior studies, a third of the standard deviation or larger is often used as a threshold for clinical significance [38, 39]. Small effects could be detected as statistically significant, and the study is powered to detect any meaningful differences between IF and IF + EF on beneficiary outcomes. The tests of mediation effects (aim 2) will have a greater power as further reduction in error variance will occur due to controlling for the mediator. Outcomes (aim 3) are benchmarked against a prior period and have no hypotheses; thus, power considerations are not applicable. Similarly, exploratory aims have no associated hypotheses.

#### Randomization

Randomization will occur at the site level. The number of beneficiaries and quality assessment scores will be used to block the 18 sites into a pair of similar attributes. A coin will be flipped to determine arm assignment of each pair. We expect to start one pair each month until all are underway.

#### Blinding

Clinicians and beneficiaries will be blinded to arm assignment. Sites, IFs, and research assistants will not be blinded. Success of blinding will be assessed at study end.

#### Study procedures

After IRB approval, data use agreements will be obtained and contracts will be executed with the sites, the state, and the EHR company. To randomize, the sites’ number of beneficiaries and summed quality data scores (2015–2017) will be obtained from the state and the investigators will match site pairs and assign the trial arm. If the clinicians consent, characteristics, ILS, ORC, GSE, and EBPAS will be collected via survey (baseline). Waiver directors and investigator will select IFs. Clinicians, IFs, and EFs will be trained. The intervention will be provided to beneficiaries and documented in the EHR (12 months post-implementation). Monthly for 11 months following implementation, the SIC (via research assistant phone interview) and intervention fidelity checklist (via project manager chart audit) will be collected. Clinician EBPAS and GSE will be collected again via survey (9 months). We will obtain beneficiary assessment data prior to the intervention (last assessment before implementation), after the intervention (first assessment within 12 months post-implementation), and any assessments that occurred between the two time points after completion of implementation via a data use agreement. Analysis will occur and reports will be written.

#### Research assistant training

Research assistants will complete human subject training and orientation prior to collecting data or interacting with IFs. As part of orientation, a job description, which includes the role and responsibilities, will be reviewed and signed; a position manual, the manual of procedures, the data safety monitoring plan and board charter, the study protocol, the literature supporting project evidence and approach, and the quality assurance-monitoring plan will be reviewed. Research assistants will conduct dummy data collection until performed correctly, prior to data collection for the trial. Once trained, the research assistant will participate in team meetings and quality assurance activities and complete a weekly report of activities and/or issues that arise.

#### Analytic approach

Preliminary analysis of the distributions of outcomes, mediators, and potential covariates will be assessed, outliers will be investigated by inspecting the residuals, and models described below will be fit with and without outliers to examine their influence on the results. Analyses will be implemented in SAS 9.4.

Aim 1 primary outcomes (adoption and sustainability) will be analyzed using a linear mixed effects model with repeated measures (SIC and EHR 12-months; IF 9-months). Covariates will include trial arm, randomization variables, and time entered as a class variable to model potentially non-linear patterns. We will use a general linear model to analyze training fidelity. Log-rank test and Cox proportional hazard modeling will be employed to analyze time spent in each implementation stage. Tests of significance of the coefficient of the trial arm variable will yield the formal test of hypothesis for the effect of IF + EF compared to IF alone. Analysis of beneficiary outcomes will employ a generalized linear model with appropriately distributed errors and the random effect of site added to account for nesting of individuals within sites. For counts of falls and health service use, Poisson error distribution will be specified. Alternatively, a zero-inflated Poisson or negative binomial model based on the distribution of the counts will be fit. Explanatory variables, including trial arm, will be evaluated as predictors of zero inflation (whether or not the count is zero) and as predictors of the magnitude of the count when it is not zero. Prior MDS will be used to obtain baseline data for each outcome, which will be included as a covariate to explain the variation in post-intervention outcomes.

Aim 2 determines whether the effects of IF + EF compared to IF alone on adoption and sustainability are mediated by clinician attitude or self-efficacy at 9 months. The model fit will be at the clinician level with site as a random effect to account for nesting of clinicians within sites. To test for mediation, trial arm will be treated as the independent variable, with each potential mediator (one at a time) tested for their effect on the outcome. A bias-corrected bootstrapping analytic strategy [40, 41] will be used to estimate confidence intervals around the indirect effect of the trial arm on the outcome variable through the mediator. To establish mediation, the 95% confidence interval around the indirect must not include 0.

Aim 3 benchmarks the effects of IF + EF compared to IF alone on beneficiary outcomes following implementation as compared to pre-intervention. Analysis will use 2 repeated measures of beneficiary outcomes, prior to and 12 months after the intervention. Time by trial arm interaction will be included. The least square means according to the interaction term will be output from this model, and differences from before and after the intervention will be evaluated for each arm to gauge the magnitude and meaning of improvements.

*Exploratory aims*. Aim 4 compares potential mediators at 9 months and baseline leadership and readiness for sites with SIC (> 50% to < 50%) at 6 months by arm. General or generalized linear models (as appropriate based on outcome or potential mediator distribution) will include the binary indicator of whether SIC at 6 months (> 50% or < 50%) in interaction with the trial arm variable. Differences between least square means according to SIC level will be tested within each arm, and the effect sizes will be estimated. Aim 5 explores whether baseline site leadership and readiness moderate the impact of IF + EF compared to IF alone on primary and secondary outcomes within 12 months in order to determine which sites may require facilitation that is more intensive. A model similar to aim 1 will be modified to include trial arm by potential moderator interaction. Effect sizes that correspond to the interaction term will be estimated. Aim 6 evaluates clinician satisfaction at 1 month and the cost of implementation and policy impact for IF + EF compared to IF alone at 12 months. We will use descriptive statistics to summarize satisfaction with training and implementation cost. We will use qualitative thematic analysis to analyze the surveys. We will report on the policy in the state contract.

## Discussion

This trial sets an ambitious agenda to explore ways to adopt and sustain evidence across a Medicaid program. There is a significant amount of work that presents challenges and opportunities. First, implementation and evaluation with a hybrid III design in an under-resourced real-world setting force a delicate balance of study design and voluntary participation. Our work with sites in a standardized manner encourages local adaptations to optimize implementation. Second, our selection of volunteer sites may limit heterogeneity in our sample. However, we expect to learn a great deal about stages of implementation to inform this project’s adoption and sustainability that may be generalizable to other settings or populations. Third, our measurement approach involves significant effort in data collection of sites, clinicians, and beneficiaries with surveys, interviews, and extraction of secondary administrative and clinical data across disparate clinician contexts. The challenges of data collection and management may be outweighed by the opportunity to generate a broader understanding in Medicaid settings to facilitate effective use of evidence in under-resourced, complex environments.

## Additional files

Additional file 1: Certification program training outline. An outline of the training modules for the certification program. (PDF 72 kb)

Additional file 2: Pocket card algorithm. A cognitive aid on a packet card that includes a set of rules the clinician will follow when examining the beneficiary’s need or potential benefit from the intervention. (PDF 114 kb)

Additional file 3: Internal facilitator (IF) training outline. An outline of the modules for the internal facilitator (IF) training program. (PDF 57 kb)

Additional file 4: Internal facilitator/external facilitator (IF/EF) audit tool. Data collection tool to examine how often the internal facilitators and external facilitators performed facilitation tasks like conducting problem solving, feedback, reflection, counseling, and remediation with a support coordinator. (PDF 56 kb)

Additional file 5: Implementation strategies and intervention fidelity audit tool. Data collection tool to examine fidelity to the implementation strategies and intervention which includes items like number of consented clinicians, completed surveys, trained clinicians, interdisciplinary meetings, and IF actions and PCSP presence of desire of beneficiary, completed SW and OT assessments and medication reviews, and if brainstorming, problem-solving, and role modeling was conducted. (PDF 49 kb)

## Abbreviations

ADL: Activities of daily living
Bb: Blackboard
EBPAS: Evidence-Based Practice Attitude Scale
ED: Emergency department
EF: External facilitation/facilitator
EHR: Electronic health record
GSE: General Self-Efficacy
IADL: Instrumental activities of daily living
IF: Internal facilitation/facilitator
ILS: Implementation Leadership Scale
MDS: Minimum Data Set for Home Care
ORCA: Organizational Readiness for Change
OT: Occupational therapist
RN: Registered nurse
SIC: Stages of Implementation Completion
SW: Social worker
