# Supplementary material for: Testing an implementation strategy bundle on adoption and sustainability of evidence to optimize physical function in community-dwelling disabled and older adults in a Medicaid waiver: a multi-site pragmatic hybrid type III protocol
Source: Implement Sci. 2019 Jun 13;14:60. doi: 10.1186/s13012-019-0907-1 (PMC6567613; doi:10.1186/s13012-019-0907-1)
Supplement: Supplementary file 1 — Certification program training outline. An outline of the training modules for the certification program. (PDF 72 kb) [file 13012_2019_907_MOESM1_ESM.pdf]

Thank you for completing the Certification Program. The program will provide knowledge regarding a new evidence-based model of care for MI Choice participants. You will learn:

1. Person-centered and self-directed care planning and provision.
2. The principles, approaches, and techniques to provide the model of care to participants.
3. RNs will learn how to review and manage medications using an App.
4. Your role and responsibilities regarding use the model of care with participants.

Over the next 30-days, take a survey, view the 20-videos, read 5-papers, and examine participant handouts. This will take approximately 4-hours and you are able to stop and restart as needed. After completing all steps below, you will receive a certificate of completion.

Folder 1 What are the core principles of an evidence-based model of care?

- Video 1 What is an evidence-based model of care?
- Video 2 Person-Environment "Fit"
- Video 3 Person-Centered Care
- Video 4 Building Collaborative Relationships
- Video 5 Building Self-Efficacy

Folder 2 What are the approaches used to conduct an evidence-based model of care?

- Video 6 Readiness to Change
- Video 7 Motivational Interviewing
- Video 8 Cognitive Behavioral Therapy
- Video 9 Problem Solving & Brainstorming

Folder 3 What are the techniques to conduct an evidence-based model of care?

- Video 10 Therapeutic Communication and Collaboration Techniques
- Video 11 Reflection
- Video 12 Inter-disciplinary Coordination

Folder 4 How to conduct the evidence-based model of care?

- Video 13 What is the role of RN, OT, and SW?
- Video 14 SW Care
- Video 15 RN Care
- Video 16 Medication Review and Management
- Video 17 Apps to Review Medications
- Video 18 OT Care
- Video 19 Case Study

Folder 5 How to implement the model of care with participants?

- Video 20 How to implement with participants?

Participants Handouts to print: Aging Toolkit; Information Sheets; Medication Calendar
Medication App: Web URL link
Articles: Bridges 2013, Szanton 2014, 2016-2, Spoelstra 2018; Beers Criteria and Essential Oils
Contact Information: Project Staff at the University and Blackboard Helpline
Reference List
