# Supplementary material for: Testing an implementation strategy bundle on adoption and sustainability of evidence to optimize physical function in community-dwelling disabled and older adults in a Medicaid waiver: a multi-site pragmatic hybrid type III protocol
Source: Implement Sci. 2019 Jun 13;14:60. doi: 10.1186/s13012-019-0907-1 (PMC6567613; doi:10.1186/s13012-019-0907-1)
Supplement: Supplementary file 2 — Pocket card algorithm. A cognitive aid on a packet card that includes a set of rules the clinician will follow when examining the beneficiary’s need or potential benefit from the intervention. (PDF 114 kb) [file 13012_2019_907_MOESM2_ESM.pdf]

Pocket Card

Determining participant need and providing the model of care

RN or SW phone call or home visit to participant:

If "YES" to questions 1-6, participant could benefit from the model of care.

1. Was participant a new admission to MI Choice?	YES	NO	N/A
2. Did the participant just get discharged (hospital/nursing home)?	YES	NO	N/A
3. Is the participant cognizant and able to communicate?	YES	NO	N/A
4. Did the participant have a desire or need?	YES	NO	N/A
5. Is the participant ready to change?	YES	NO	N/A
6. Is the participant motivated to change?	YES	NO	N/A

If "YES" to questions 7-10, may need OT or increased RN or SW home visits briefly.

7. Is there a need for the OT to assist with function?	YES	NO	N/A
8. Does the participant have DME they do not know how to use?	YES	NO	N/A
9. Does the RN need to review the medication list?	YES	NO	N/A
10. Does the SW need to evaluate the participant's depression?	YES	NO	N/A

SC decision after considering above questions:

- Participant has a desire or need that the model of care is likely to improve
- Participant would benefit from additional home visits

Recommendation for additional home visit(s):

- OT to conduct an assessment to determine how to improve function
- RN to evaluate medications, and/or manage pain, incontinence, or other health needs
- SW to evaluate depression and/or assist with community resources

***Tailor PCSP** Add the model of care to Arranged Services

**Additional Home Visits**	**OT 1 to 6**	Assessment and observation, conduct safety tour and brainstorm how to improve function, working with participant to problem solve
	**RN 1 to 4**	Review medications using App and modify regimen as needed, and/or brainstorm how to manage pain, incontinence, or other needs, working with participant to problem solve<br>Provide Toolkit and fall prevention materials
	**SW 1 or 2**	Brainstorm how to manage depression and/or assist with community resources, working with participant to problem solve

****Coordinate Care** OT-RN-SW jointly as needed (in person, phone, or email)

Supplies, DME, Home Modification per usual process at waiver agency

Document per usual process in assessment(s), PCSP, or PN (COMPASS or electronic record)
