# Supplementary material for: Testing an implementation strategy bundle on adoption and sustainability of evidence to optimize physical function in community-dwelling disabled and older adults in a Medicaid waiver: a multi-site pragmatic hybrid type III protocol
Source: Implement Sci. 2019 Jun 13;14:60. doi: 10.1186/s13012-019-0907-1 (PMC6567613; doi:10.1186/s13012-019-0907-1)
Supplement: Supplementary file 3 — Internal facilitator (IF) training outline. An outline of the modules for the internal facilitator (IF) training program. (PDF 57 kb) [file 13012_2019_907_MOESM3_ESM.pdf]

Facilitator Training Outline

Supervisors will complete the Facilitator Training program over 15-days by viewing the 10 to 15 minute videos (approximately 1-2 hours) to understand how to implement the new model of care.

Video 1	Role and Responsibilities of the Supervisor to Implement Model of Care
Video 2	Overview of Evidence-based Techniques
Video 3	Quality Improvement: What is it?
Video 4	How to Problem Solve
Video 5	How to provide Feedback
Video 6	How to promote Reflection
Video 7	How to provide Counseling
Video 8	How to use Motivational Interviewing
Video 9	How to provide Remediation
Video 10	The Implementation Plan

Materials to Download and Print for use with Supports Coordinators:

1. Implementation Toolkit
2. Posters
3. Pocket Card
