# Supplementary material for: Testing an implementation strategy bundle on adoption and sustainability of evidence to optimize physical function in community-dwelling disabled and older adults in a Medicaid waiver: a multi-site pragmatic hybrid type III protocol
Source: Implement Sci. 2019 Jun 13;14:60. doi: 10.1186/s13012-019-0907-1 (PMC6567613; doi:10.1186/s13012-019-0907-1)
Supplement: Supplementary file 4 — Internal facilitator/external facilitator (IF/EF) audit tool. Data collection tool to examine how often the internal facilitators and external facilitators performed facilitation tasks like conducting problem solving, feedback, reflection, counseling, and remediation with a support coordinator. (PDF 56 kb) [file 13012_2019_907_MOESM4_ESM.pdf]

Internal Facilitator/ External Facilitator (IF/EF) Audit Tool

Questions		Answer
Start Time:		
Date:		
Interviewer Name:		
Waiver Site:		
Interviewee (IF/Champion) Name:		
Are you a new IF/Champion? If yes, do next question. If no, skip next question		
How long have you been an IF/Champion?		
On a scale of 0=none to 10=all the time, how much did you:	Talk face-to-face with a Supports Coordinator?	
	Review QI data?	
	Talk to a Supports Coordinator about QI data?	
	Conduct problem solving with a Supports Coordinator?	
	Conduct feedback with a Supports Coordinator?	
	Conduct reflection with a Supports Coordinator?	
	Conduct counseling with a Supports Coordinator?	
	Conduct remediation with a Supports Coordinator?	
	Use Motivational Interviewing with a Supports Coordinator?	
	Talk to a Supports Coordinator about completing certification?	
	Examine COMPASS documentation for CAPABLE?	
Over the past month, how many times did:	Talk with other IFs?<br>The EF contact you?<br>What was the nature of your discussion?	
	Did you contact the EF?<br>What was the nature of your discussion?	
On a scale of 1 to 10 with 1 being none to 10 being a lot, how well did the assistance from the EF guide your work?		
IS there anything else you would like to tell me?		
End Time:		
