# Supplementary material for: Testing an implementation strategy bundle on adoption and sustainability of evidence to optimize physical function in community-dwelling disabled and older adults in a Medicaid waiver: a multi-site pragmatic hybrid type III protocol
Source: Implement Sci. 2019 Jun 13;14:60. doi: 10.1186/s13012-019-0907-1 (PMC6567613; doi:10.1186/s13012-019-0907-1)
Supplement: Supplementary file 5 — Implementation strategies and intervention fidelity audit tool. Data collection tool to examine fidelity to the implementation strategies and intervention which includes items like number of consented clinicians, completed surveys, trained clinicians, interdisciplinary meetings, and IF actions and PCSP presence of desire of beneficiary, completed SW and OT assessments and medication reviews, and if brainstorming, problem-solving, and role modeling was conducted. (PDF 49 kb) [file 13012_2019_907_MOESM5_ESM.pdf]

Implementation Strategies and Intervention Fidelity Audit Tool

Relationship building	# of contracts (sites)<br># of consents (clinicians)<br># of DUA (CIM/sites/MDHHS)
Readiness, leadership, attitude assessment	yes/no
Champion building	# of IFs
IF Actions	training, coaching, consultation, supervision, modeling, problem solving, and providing feedback, supporting, instructing, demonstrating, and assisting with evaluation
Clinician certification	yes/no
Interdisciplinary Coordination	PN#
OT Assessment	yes/no
SW mood Assessment	yes/no
RN medication review	yes/no
PSCP presence of desire of beneficiary	yes/no
PN brainstorming, problem solving, role modeling	yes/no
Cost	Time $ calculation
Policy	yes/no
